# Erastin Disrupts Mitochondrial Permeability Transition Pore (mPTP) and Induces Apoptotic Death of Colorectal Cancer Cells

**DOI:** 10.1371/journal.pone.0154605

**Published:** 2016-05-12

**Authors:** Haizhong Huo, Zhiyuan Zhou, Jian Qin, Wenyong Liu, Bing Wang, Yan Gu

**Affiliations:** Department of General Surgery, The Ninth People's Hospital Affiliated to Shanghai Jiao-Tong University School of Medicine, Shanghai, China; Suzhou University, CHINA

## Abstract

We here evaluated the potential anti-colorectal cancer activity by erastin, a voltage-dependent anion channel (VDAC)-binding compound. Our *in vitro* studies showed that erastin exerted potent cytotoxic effects against multiple human colorectal cancer cell lines, possibly via inducing oxidative stress and caspase-9 dependent cell apoptosis. Further, mitochondrial permeability transition pore (mPTP) opening was observed in erastin-treated cancer cells, which was evidenced by VDAC-1 and cyclophilin-D (Cyp-D) association, mitochondrial depolarization, and cytochrome C release. Caspase inhibitors, the ROS scavenger MnTBAP, and mPTP blockers (sanglifehrin A, cyclosporin A and bongkrekic acid), as well as shRNA-mediated knockdown of VDAC-1, all significantly attenuated erastin-induced cytotoxicity and apoptosis in colorectal cancer cells. On the other hand, over-expression of VDAC-1 augmented erastin-induced ROS production, mPTP opening, and colorectal cancer cell apoptosis. *In vivo* studies showed that intraperitoneal injection of erastin at well-tolerated doses dramatically inhibited HT-29 xenograft growth in severe combined immunodeficient (SCID) mice. Together, these results demonstrate that erastin is cytotoxic and pro-apoptotic to colorectal cancer cells. Erastin may be further investigated as a novel anti-colorectal cancer agent.

## Introduction

The colorectal cancer is the major contributor of cancer-related mortality both in China [1] and around the world [2,3]. It is estimated that over 100,000 new cases of colorectal cancer are diagnosed each year, which cause over 50,000 deaths annually [4]. Chemotherapy has been widely-utilized for treatment of colorectal cancer, however drug resistance and/or off-target toxicity limit the efficiency of current chemo-drugs [5,6,7]. Thus, our group [8,9] and others [10,11] have been focusing on the development of novel and more efficient anti-colorectal cancer agents.

Mitochondrial permeability transition pore (mPTP) is a multi-protein channel complex lying in the mitochondria, whose main function is to maintain the balance of mitochondrial respiratory chain [12]. mPTP is primarily composed of three proteins: including voltage-dependent anion channel (VDAC) in the out mitochondrial membrane (OMM), adenine nucleotide translocator 1 (ANT-1) in the inner mitochondrial membrane (IMM) and matrix locating cyclophilin-D (Cyp-D) [12]. It has been shown that multiple stimuli will induce ANT-1 and Cyp-D association and mPTP opening, thus leading to reactive oxygen species (ROS) production, ATP depletion and pro-apoptotic molecule (*i*.*e*. cytochrome c) release [12,13]. Thereafter, caspases (mainly caspase-9) and cell apoptosis will be activated [12,13].

Recent studies have identified a first-in-class VDAC-binding small molecule, namely erastin [14,15]. It has been shown that erastin is selectively cytotoxic to certain cancer cell lines [14,15,16,17]. For example, Yagoda et al., showed that erastin binds to VDAC, resulting in lethal oxidative damage to cancer cells [18]. The potential role of erastin in colorectal cancer cells, and underlying signaling mechanisms have not been studied. In the current study, we showed that erastin was cytotoxic and pro-apoptotic to colorectal cancer cells, possibly via disrupting mPTP.

## Materials and Methods

### 2.1. Cell culture

As described [8,9], colorectal cancer cell lines, including HT-29, DLD-1 and Caco-2, were purchased from the cell bank of Chinese Academy of Science (CAS) Shanghai Biological Institute (Shanghai, China). Cells were maintained in FBS-containing RPMI/DMEM medium. Human NCM460 colon epithelial cell line was provided by Fudan IBS Cell center (Shanghai, China). Cells were cultured in Ham's F12 nutrient medium (Gibco) [19].

### 2.2. Reagents and chemicals

Erastin was purchased from Selleck (Shanghai, China). The mPTP blockers including sanglifehrin A, cyclosporin A and bongkrekic acid were purchased from Sigma (Shanghai, China). The anti-oxidant MnTBAP was also from Sigma. The caspase-3 specific inhibitor z-DEVD-fmk and the caspase-9 specific inhibitor z-LEHD-fmk were purchased from Calbiochem (Darmstadt, Germany). All antibodies applied in this study were obtained from Santa Cruz Biotech (Shanghai, China).

### 2.3. MTT cell viability assay

Cell survival was measured by the 3-[4,5-dimethylthylthiazol-2-yl]-2,5 diphenyltetrazolium bromide (MTT, Sigma) assay [9]. Different seeding densities were optimized at the beginning of the experiments.

### 2.4. Trypan blue staining assay

Trypan blue assay was described in our previous studies [9]. Briefly, after applied erastin treatment, the number of dead (trypan blue positive) cells was counted. The death ratio (%) was calculated by the number of the trypan blue stained cells divided by the total number of the cells.

### 2.5. Colony formation assay

Following the erastin treatment, cells (2 × 10^3^) were initially suspended in culture medium with 0.25% agar (Sigma). The cell suspension was then planked on top of a pre-solidified 0.25% agar onto a 100-mm culture dish. The medium was replaced every two days. After 10 days of incubation, survival colonies were stained and manually counted.

### 2.6. BrdU incorporation assay

Cells (3×10^3^ per well) were seeded onto 96-well plates, after applied treatment, cell proliferation was assessed via the BrdU incorporation ELISA colorimetric assay (Roche, Indianapolis, IN) with the manufacturer’s protocol. The ELISA OD value of treatment group was normalized to that of untreated control group.

### 2.7. Cell apoptosis assay through Annexin V staining

After treatment, cell apoptosis was detected by the Annexin V FACS assay as previously reported [9]. The number of Annexin V stained cells was recorded.

### 2.8. Quantification of apoptosis by enzyme-linked immunosorbent assay (ELISA)

As described [8,9], the Cell Apoptosis ELISA Detection Kit Plus (Roche, Palo Alto, CA) was utilized to quantify cell apoptosis according to the manufacturer's protocol.

### 2.9. Caspase activity assay

After treatment, cytosolic proteins were extracted in the buffer described [8,9]. Twenty μg of cytosolic extracts per sample were added to caspase assay buffer with substrates of caspase-3/-8/-9 (Roche, Shanghai, China). The release of 7-amido-4-(trifluoromethyl)coumarin (AFC) was quantified via a Fluoroskan system set to an excitation value of 355 nm [8,9]. The results were expressed as relative fluorescence units/μg of protein.

### 2.10. Western blotting

Briefly, the aliquots of 30 μg of lysed proteins of each sample were separated by 10% SDS polyacrylamide gel electrophoresis and transferred onto PVDF membranes (Millipore, Bedford, MA). After blocking, the membranes were incubated with the primary antibody overnight at 4°C, followed by incubation with secondary antibody for one hour at room temperature. The bolt was visualized by ECL (enhanced chemiluminescence) machine. Each band was quantified via ImageJ software, and the value was normalized to each loading control band.

### 2.11. Reactive oxygen species (ROS) detection

Intracellular ROS was measured by flow cytometry via dichlorofluorescin (DCF) oxidation assay. DCFH-DA enters passively into cells and is cleaved by nonspecific cellular esterases and oxidized in the presence of ROS. After treatment, cells (3×10^5^ per sample) were incubated with DCFH-DA (5 μM) for one hour at 37°C. Thereafter, cells were washed with PBS and kept in 1 mL of PBS, ROS fluorescence was analyzed using the above Fluoroskan system.

### 2.12. Detection of mitochondrial potential reduction (ΔΨ_m_)

As described [20], the ΔΨ_m_ was measured through JC-10 fluorescence dye. When the mitochondrial potential is decreasing, monomeric JC-10 will form in the cytosol, which exhibits green fluorescence [20]. Briefly, after applied erastin treatment, cells were stained with 5 μg/mL of JC-10 (Invitrogen) for 10 min, and detected immediately on a Fluoroskan system set to an excitation value of 485 nm [20].

### 2.13. Mitochondrial immunoprecipitation (mito-IP)

Cells were trypsinized, and mitochondrial fractions were prepared via a Mitochondria/Cytosol Fractionation Kit (BioVision, Shanghai, China) according to the manufacturer's instructions. Two hundred μg of cell lysates from mitochondrial fractions were pre-cleared with 20 μL of protein A/G PLUS-agarose (Santa Cruz) for 1 hour. The supernatant was then rotated overnight with 0.25 μg of anti-ANT-1 (Santa Cruz Biotech). Next, the lysates were centrifuged for 5 min at 4°C in a micro-centrifuge to remove nonspecific aggregates. The protein A/G PLUS-agarose (35 μL) was then added to the supernatants for 4 hours at 4°C. Pellets were washed six times with PBS, resuspended in lysis buffer, and then assayed by Western blotting [20].

### 2.14. Stable knockdown of VDAC-1 by lentiviral shRNA

The two sets of lentivirus-packaged VDAC-1 short hairpin RNAs (shRNA-1 and shRNA-2, non-overlapping sequences) were designed, synthesized and verified by Genechem (Shanghai, China). Ten μL/mL of lentiviral particles were added to HT-29 cells for 12 hours. Afterwards, lentivirus containing medium was replaced by complete medium, and cells were cultured for another 24 hours. Afterwards, puromycin (5.0 μg/mL, Sigma) was added to select resistant stably colonies for 2–3 weeks. Expression of VDAC-1 was detected by Western blotting. Control cells were treated with scramble non-sense shRNA lentiviral particles (Santa Cruz Biotech).

### 2.15 Over-expression of VDAC-1 and stably cells selection

The full-length human VDAC-1 cDNA, purchased from Genechem (Shanghai, China), was sub-cloned into pSuper-puro-flag (a gift from Dr. Bi’s Lab)[21]. The empty vector (pSuper-puro-flag) or VDAC-1 expressing construct was transfected into HT-29 cells via Lipofectamine 2000 protocol (Invitrogen). The stably clones were selected via puromycin (5 μg/mL). After 12–14 days of selection, stably cells were subjected to Western blotting assay of VDAC-1 expression.

### 2.16. *In vivo* antitumor efficacy evaluation

Tumor growth studies were performed in severe combined immunodeficient (SCID) mice xenograft model. All mice were purchased from the Animal Facility of Shanghai Jiao-tong University School of Medicine (Shanghai, China). Briefly, 2×10^6^ viable HT-29 cells in 100 μL of growth medium (per mouse) were subcutaneously inoculated, and mice bearing ~100 mm^3^ tumors were randomly divided into three groups with 10 mice per group. Mice were treated daily with 10 or 30 mg/kg body weight of erastin (intraperitoneal injection, for 4 weeks) or vehicle control (Saline). Tumor volumes were calculated by the modified ellipsoid formula: (π / 6) ×AB^2^, where A is the longest and B is the shortest perpendicular axis of a tumor mass [22,23]. Mice body weights were also recorded every week. Humane endpoints were always utilized to minimize mice suffering. Animals were observed on daily bases. Signs such as significant-reduced locomotion, severe diarrhea, severe piloerection or a sudden weight loss (> 20%) were recorded. If animals reached these endpoints they were euthanized by exsanguination under 2,2,2-tribromoethanol anesthesia (4 mg/10 g body weight, Sigma). All injections were performed under the 2,2,2-tribromoethanol anesthesia method. The animal studies have been approved by the Shanghai Jiao-tong University School of Medicine’s Institutional Animal Care and Use Committee (IACUC) and Ethics committee (Contact person: Dr. Jun Wang, 2014126).

### 2.17. Statistical analysis

All data were normalized to control values of each assay and were presented as mean ± standard deviation (SD). Data were analyzed by one-way ANOVA followed by a Scheffe’s f-test by using SPSS 16.0 software (SPSS Inc., Chicago, IL). Significance was chosen as **p** < 0.05.

## Results

### 3.1. Erastin exerts cytotoxic, but not cytostatic effects to cultured colorectal cancer cells

**Fig 1:**
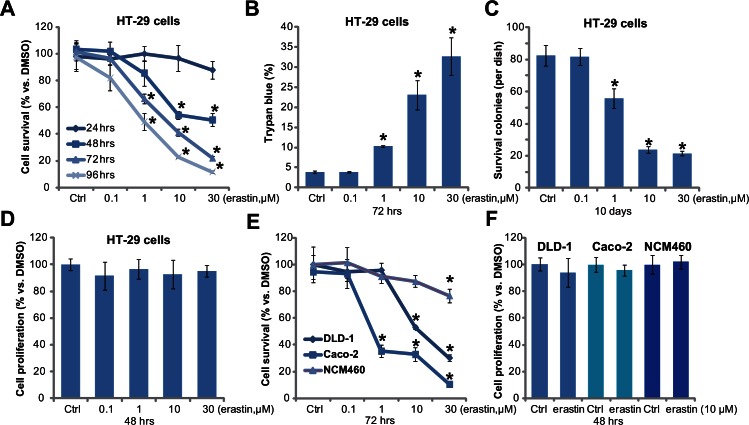
Erastin exerts cytotoxic, but not cytostatic, effects to cultured colorectal cancer cells. Colorectal cancer cells (HT-29, DLD-1 and Caco-2 lines) or NCM460 colon epithelial cells were treated with vehicle control (0.1% DMSO, “Ctrl”) or indicated concentrations of erastin for applied time, cell survival was tested by MTT assay (A and E) and colony formation assay (C); The percentage of trypan blue positive (“dead” cells) was recorded (B); Cell proliferation was tested by BrdU incorporation assay (D and F). For each assay, n = 5. The data presented were mean ± SD. Experiments were repeated three times with similar results obtained. * p < 0.05 vs. group of “Ctrl”.

To test erastin’s activity on colorectal cancer cell survival, HT-29 cells were treated with increasing concentrations of erastin (0.1–30 μM). MTT assay was performed. As shown in Fig 1A, erastin potently inhibited HT-29 cell survival, which was evidenced by MTT OD reduction. Erastin showed a dose-dependent effect (Fig 1A), and 30 μM of erastin displayed the most dramatic effect (Fig 1A). Erastin took at least 48 hours to exert significant cytotoxic effect in HT-29 cells (Fig 1A). The cytotoxic effect by erastin was also demonstrated by the trypan blue staining assay (Fig 1B) and colony formation assay (Fig 1C). Erastin (1–30 μM) treatment significantly increased the number of trypan blue positive (“dead”) HT-29 cells (Fig 1B), whiling decreasing survival HT-29 colonies (Fig 1C).

Interestingly, erastin (1–30 μM) appeared ineffective in inhibiting HT-29 cell proliferation, and the BrdU incorporation was not changed in HT-29 cells after cytotoxic erastin (1–30 μM) treatment (Fig 1D). Therefore, the cytotoxic effect by erastin is unlikely due to proliferation inhibition. MTT results in Fig 1E showed that erastin (1–30 μM) was also cytotoxic to two other colorectal cancer cell lines: DLD-1 and CaCo2. Yet, same erastin treatment was generally safe to the non-cancerous NCM460 colon epithelial cells (Fig 1E). Once again, BrdU incorporation, the indicator of cell proliferation, was not affected by erastin (10 μM) in the DLD-1 and CaCo2 cells, nor in NCM460 epithelial cells (Fig 1F). Based on these results, we show that erastin exerts cytotoxic, but not cytostatic, activity to cultured colorectal cancer cells.

### 3.2. Erastin induces ROS production and caspase-dependent apoptosis in cultured colorectal cancer cells

**Fig 2:**
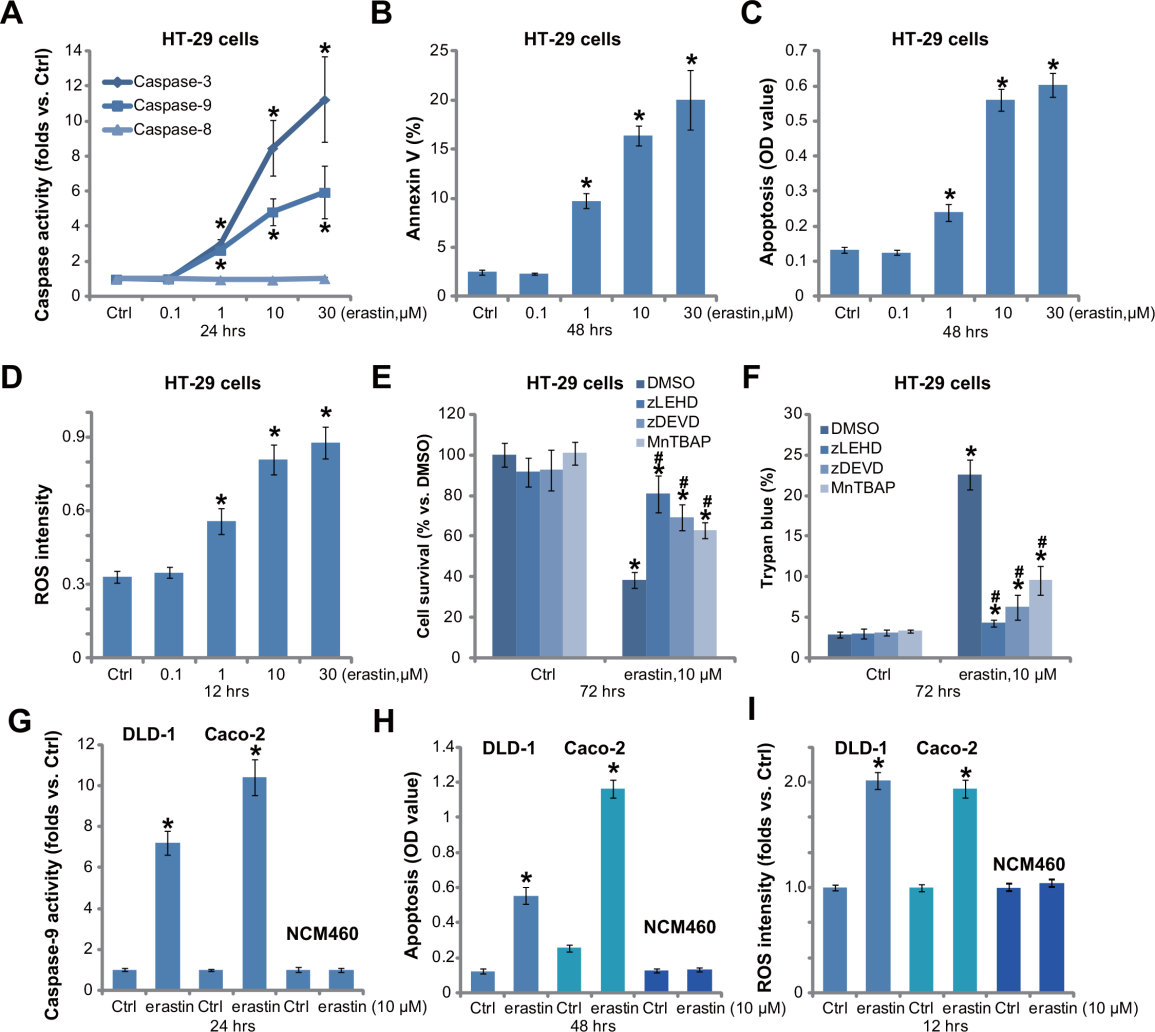
Erastin induces ROS production and caspase-dependent apoptosis in cultured colorectal cancer cells. Colorectal cancer cells (HT-29, DLD-1 and Caco-2 lines) or NCM460 colon epithelial cells were treated with vehicle control (0.1% DMSO, “Ctrl”) or indicated concentrations of erastin for applied time, cell apoptosis was examined by listed assays (A-C, G and H); ROS production was also examined (D and I). HT-29 cells were pre-treated with z-DEVD-fmk (“zDEVD”, 50 μM), z-LEHD-fmk (“zLEHD”, 50 μM) or MnTBAP (10 μM) for 1 hour prior to applied erastin stimulation, cell survival and cell death were tested by MTT assay (E) and trypan blue assay (F), respectively. For each assay, n = 5. The data presented were mean ± SD. Experiments were repeated three times with similar results obtained. * p < 0.05 vs. group of “Ctrl”. ^#^ p < 0.05 vs. group of erastin only (E and F).

Next, we evaluated the potential activity of erastin on cell apoptosis. As described in our previous studies [8,9], various apoptosis assays were performed. The caspase assay results showed that the activity of caspase-3 and caspae-9 was significantly increased in HT-29 cells after cytotoxic erastin (1–30 μM) treatment (Fig 2A), indicating mitochondrial apoptosis pathway activation [24]. On the other hand, the activity of caspase-8, an indicator of extrinsic apoptotic pathway activation [25,26], was unchanged in erastin-treated HT-29 cells (Fig 2A). Cell apoptosis activation by erastin was also confirmed by the Annexin V FACS assay (Fig 2B) and Histone DNA apoptosis ELISA assay (Fig 2C). Erastin dose-dependently increased Annexin V percentage and Histone DNA ELISA OD in HT-29 cells (Fig 2B and 2C).

Since erastin is a VDAC-binding compound, which may disrupt mitochondrial respiratory chain and cause ROS production [18]. We next tested the oxidative stress level in erastin-treated HT-29 cells. Results in Fig 2D demonstrated clearly that erastin increased the level of ROS in HT-29 cells. To study the role of apoptosis and ROS production in erastin-induced cytotoxicity, various pharmacological inhibitors were applied. As shown in Fig 2E and 2F, the caspase-3 specific inhibitor z-DEVD-fmk, the caspase-9 specific inhibitor z-LEHD-fmk, or the superoxide scavenger MnTBAP [27] all alleviated erastin-induced cytotoxicity in HT-29 cells (Fig 2E and 2F). In two other colorectal cancer cell lines, erastin (10 μM) also induced caspase-9 (Fig 2G) and apoptosis (Fig 2H) activation as well as ROS production (Fig 2I). Such effects by erastin were again not seen in colon epithelial NCM460 cells (Fig 2G–2I). Therefore, erastin induces ROS production and caspase-dependent apoptosis in colorectal cancer cells.

### 3.3. Erastin induces mPTP opening in cultured colorectal cancer cells

**Fig 3:**
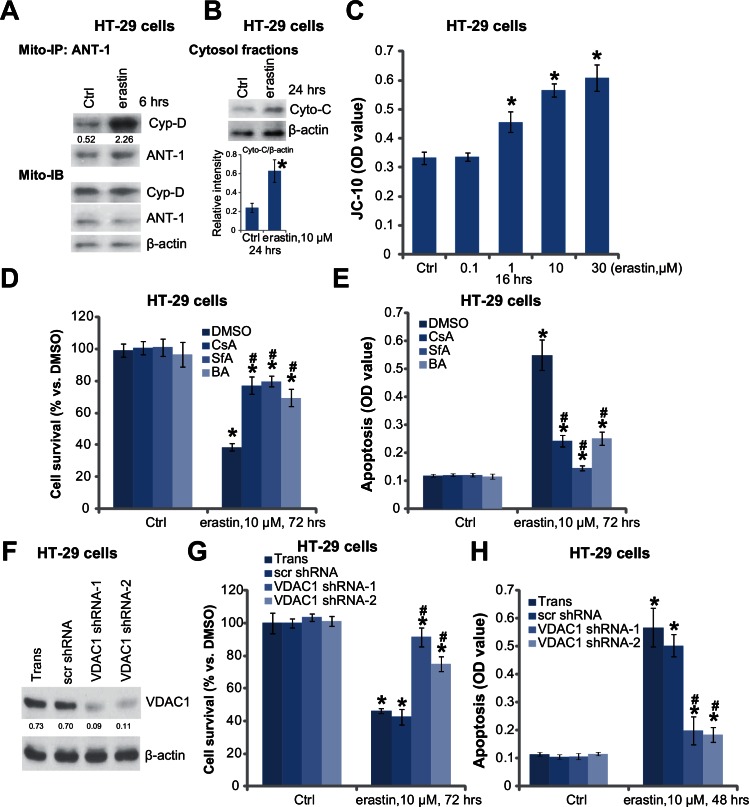
Erastin induces mPTP opening in cultured colorectal cancer cells. HT-29 cells were treated with applied erastin for indicated time, mPTP opening was evidenced by mitochondrial VDAC-1-ANT-1 association (A), cytochrome C (“Cyto-C”) release (B) and JC-10 intensity increase (C). HT-29 cells were pre-treated with sanglifehrin A (SfA, 2.5 μM), cyclosporin A (CsA, 0.5 μM) or bongkrekic acid (BA, 5 μM) prior to erastin (10 μM) treatment, cell survival (D) and apoptosis (E) were analyzed afterwards. Stably HT-29 cells expressing VDAC-1 shRNA-1/-2 or scramble control shRNA (“scr shRNA”) were treated with erastin (10 μM), VDAC-1 expression (F), cell survival (G) and apoptosis (H) were tested. ANT-1-assocaited Cyp-D (A), cytosol cytochrome C expression (B) and VDAC-1 expression (F) were quantified. For each assay, n = 4. The data presented were mean ± SD. Experiments were repeated three times with similar results obtained. * p < 0.05 vs. group of “Ctrl”. ^#^ p < 0.05 vs. group of erastin only (D and E) or the “scr shRNA” group (G and H). “Trans” stands for transfection control (F-H).

Since erastin is a VDAC-binding compound [18], the status of mPTP in erastin-treated colorectal cancer cells was then assessed. First, mitochondrial immunoprecipitation (Mito-IP) assay [28,29] demonstrated that ANT-1 and Cyp-D formed a complex in erastin-treated HT-29 cells (Fig 3A), which is known as the initial step of mPTP opening [12,13]. Second, the level of cytosol cytochrome C was also increased in HT-29 cells after erastin treatment (Fig 3B), which is a known event following mPTP opening [12,13]. Further, the increase of JC-10 green fluorescence intensity indicated loss of mitochondrial potential (ΔΨm) (Fig 3C). All these results clearly indicated that mPTP opening following erastin treatment in HT-29 cells. Note that similar results by erastin were also obtained in other two colorectal cancer cell lines (Data not shown).

To study the role of mPTP in erastin-caused cytotoxicity, we applied several known pharmacological mPTP blockers, including sanglifehrin A (SfA) [30], cyclosporin A (CsA) [31] and bongkrekic acid (BA) [28,32]. As demonstrated, pre-treatment with these mPTP blockers significantly attenuated erastin-induced HT-29 cell death (Fig 3D) and apoptosis (Fig 3E). To further support our hypothesis, shRNA strategy was applied to selectively and stably knockdown VDAC-1, the key component of mPTP and binding protein of erastin [12]. Two stably HT-29 lines expressing distinct VDAC-1 shRNAs (-1/-2) were established (Fig 3F). Importantly, erastin-induced cytotoxicity (Fig 3G) and apoptosis (Fig 3H) were significantly inhibited in VDAC-1-silenced HT-29 cells. These pharmacological and genetic evidences suggest that erastin-induced cytotoxicity against colorectal cancer cells requires VDAC-1 binding and subsequent mPTP opening.

### 3.4. VDAC-1 over-expression potentiates erastin’s cytotoxicity

**Fig 4:**
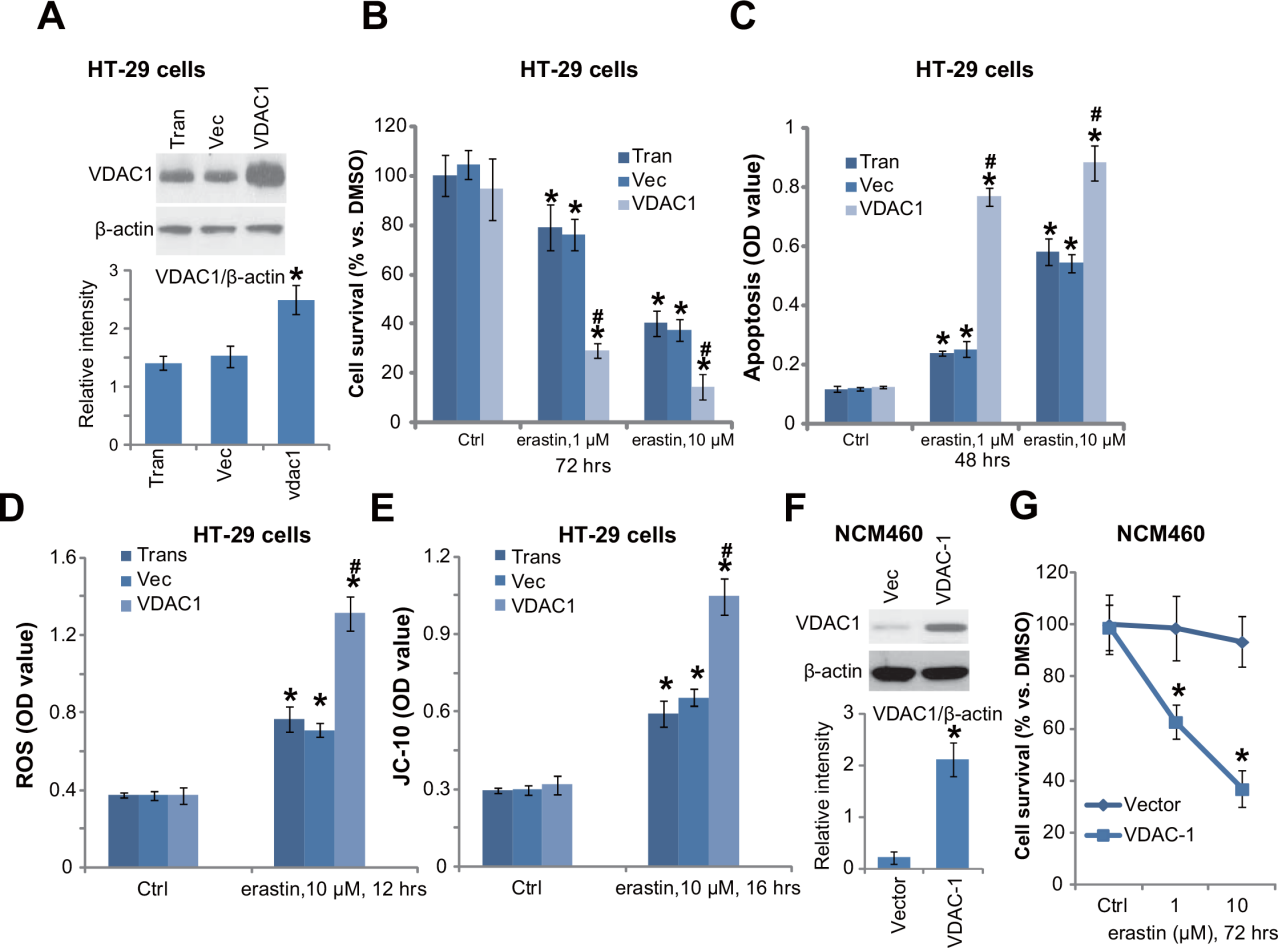
VDAC-1 over-expression potentiates erastin’s cytotoxicity. Stably HT-29 cells or NCM460 colon epithelial cells expressing empty vector (pSuper-puro, “Vec”) or VDAC-1 cDNA (“VDAC-1”) were treated with designed erastin for applied time, VDAC-1 expression, cell survival and apoptosis were tested by Western blotting assay (A and F), MTT assay (B and G) and histone DNA ELISA assay (C), respectively; ROS production (D) and JC-10 intensity (E) were also analyzed. For each assay, n = 4. The data presented were mean ± SD. Experiments were repeated three times with similar results obtained. VDAC-1 expression was quantified (F).* p < 0.05 vs. Ctrl group of “Vec” cells (B-E). ^#^ p < 0.05 vs. erastin group of “Vec” cells (B-E). “Trans” stands for transfection control (D and E).

To further support the key role of VDAC-1 in erastin-induced cytotoxicity, we exogenously expressed VDAC-1 in HT-29 cells. Western blotting results in Fig 4A confirmed VDAC-1 over-expression in stably HT-29 cells with VDAC-1 construct. As a result, erastin-induced viability reduction (Fig 4B) and apoptosis (Fig 4C) were augmented. Further studies showed that over-expression of VDAC-1 facilitated erastin-induced ROS production (Fig 4D) and JC-10 OD increase (the indicator of mPTP opening, Fig 4E). Interestingly, we showed that NCM460 colon epithelial cells expressed low level of VDAC-1 (Fig 4F). Yet, when we over-expressed VDAC-1 in NCM460 cells (Fig 4F), these cells became vulnerable to erastin (Fig 4G). Therefore, these results further confirm that VDAC-1 is a key determinant of erastin’s activity.

### 3.5. Erastin administration suppresses HT-29 xenograft growth in SCID mice

**Fig 5:**
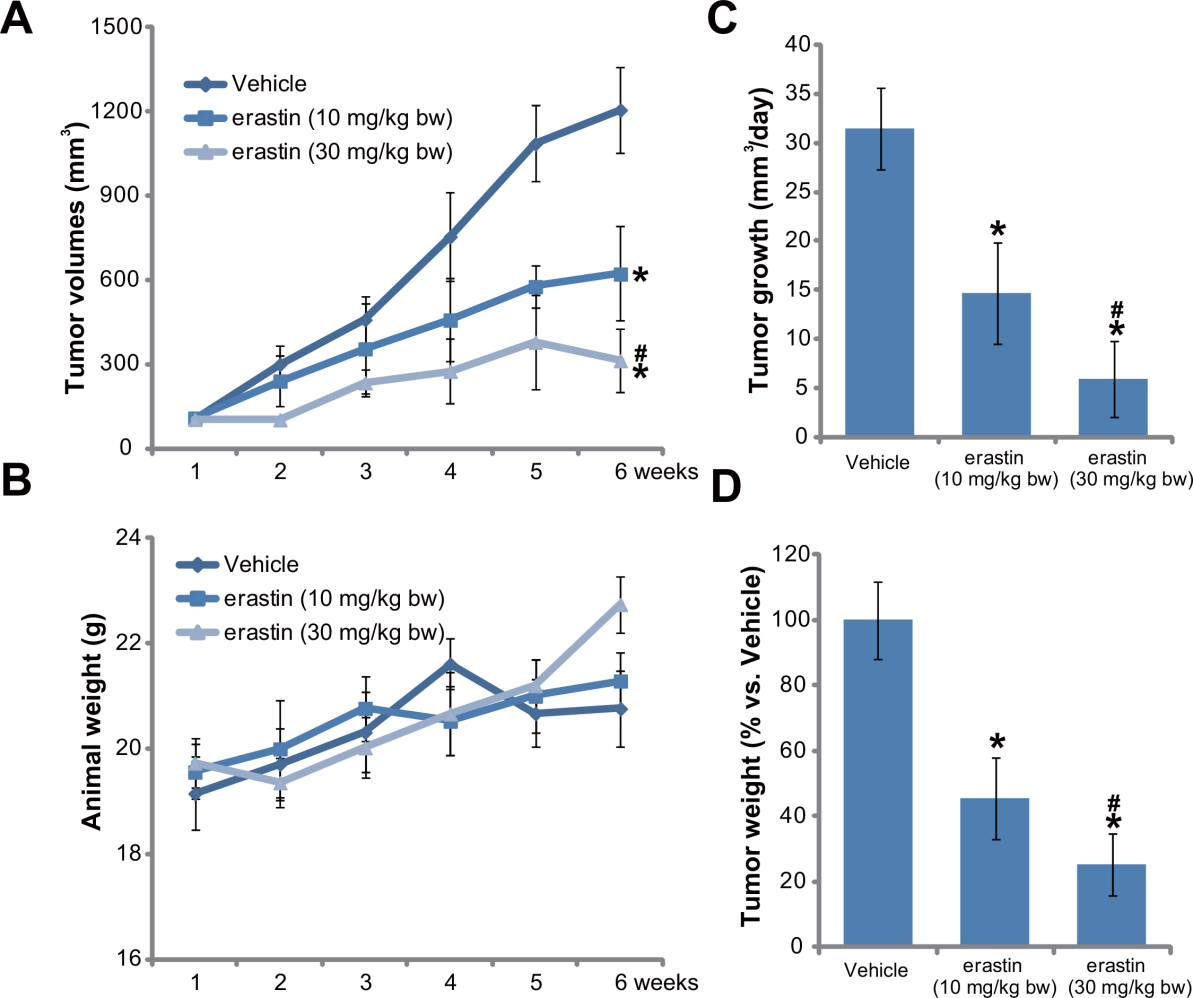
Erastin administration inhibits HT-29 xenograft growth in SCID mice. HT-29 tumor bearing SCID mice were intraperitoneally administrated with erastin (10/30 mg/kg body weight or “bw”, daily, for 4 weeks) or vehicle (Saline) control, tumor volumes (A) and mice body weights (B) were recorded weekly for five weeks; Daily tumor growth was also calculated (C). At the termination of experiments, all the xenografts were isolated and weighted (D). For each assay, n = 10. The data presented were mean ± SD. Experiments were repeated twice with similar results obtained. * p < 0.05 vs. group of “Vehicle”. ^#^ p < 0.05 vs. group of erastin at 10 mg/kg of body weight.

The *in vivo* activity by erastin was also tested. SCID mice HT-29 xenograft model was applied. The weekly tumor growth curve results in Fig 5A demonstrated that erastin intraperitoneal injection dramatically inhibited HT-29 xenograft growth in SCID mice. Erastin’s *in vivo* activity was again concentration dependent. Erastin at 30 mg/kg was clearly more potent than 10 mg/kg in suppressing HT-29 xenografts (Fig 5A). It should be noted that the mice body weight was not significant different between each groups (Fig 5B). Neither did we notice any signs of apparent toxicities in these mice. These results indicated that these animals were well-tolerated to the erastin regimens here. Further, tumor daily growth, calculated as mm^3^/day, was decreased with erastin administration (Fig 5C). At the end of experiments, the weights of erastin-administrated xenografts were also much lower than that of vehicle control mice (Fig 5D). These results showed that erastin administration inhibits HT-29 tumor growth *in vivo*.

## Discussion

In the present study, we demonstrated that erastin exerted potent cytotoxic effect against multiple human colorectal cancer cells possibly via inducing oxidative stress and caspase-9 dependent cell apoptosis. mPTP opening was observed in erastin-treated cancer cells, which was evidenced by VDAC-1 and Cyp-D association, mitochondrial depolarization and cytochrome C release. Caspase inhibitors, the ROS scavenger MnTBAP, and mPTP blockers (sanglifehrin A, cyclosporin A and bongkrekic acid), as well as shRNA-mediated knockdown of VDAC-1, all significantly attenuated erastin-induced cytotoxicity and apoptosis in colorectal cancer cells. On the other hand, over-expression of VDAC1 potentiated erastin’s cytotoxicity. Based on these results, we propose that erastin binds to VDAC1 to disrupt normal mitochondrial function, causing mPTP opening, and eventually leading to caspase-9-dependent apoptosis activation in colorectal cancer cells.

It should be noted that erastin was non-cytotoxic to NCM460 colon epithelial cells. We also failed to detect any significant caspase-9 activation nor mPTP dysfunction in erastin-treated NCM460 cells. One possible reason could be that these non-cancerous epithelial cells express very low level of VDAC (-1), therefore cells were not targeted by erastin (see Fig **4**). As a matter of fact, when we exogenously over-expressed VDAC-1 in NCM460 cells, these cells, just like cancerous cells, became vulnerable to erastin (see Fig **4**). Another possibility is that the cancerous cells are all rapid growing cells, who possibly require high level of mitochondrial respiratory chain to produce ATP. These cells might be more sensitive to VDAC or mPTP disruption. The fact that erastin only targets cancerous cells indicates that it could be an ideal candidate for anti-cancer treatment.

Although several studies have investigated the potential anti-cancer activity by erastin *in vitro* [14,16,33], the *in vivo* evidences are still lacking. In the present study, we showed that intraperitoneal injection of erastin at well-tolerated doses (10 or 30 mg/kg, daily) dramatically inhibited HT-29 xenograft growth in SCID mice. Importantly, the *in vivo* erastin regimens didn’t affect mice weights, nor did it induce any significant toxicities to the experimental animals. These preclinical results suggest that erastin could be a promising anti-colorectal cancer agent.

## Conclusions

In summary, erastin disrupts mPTP and induces apoptotic death of colorectal cancer cells. Erastin may be further investigated as a novel anti-colorectal cancer agent.

## References

[1] Chen W, Zheng R, Baade PD, Zhang S, Zeng H, Bray F, et al (2016) Cancer statistics in China, 2015. CA Cancer J Clin. 10.3322/caac.21338 26808342

[2] Chen A, Xu J, Johnson AC (2006) Curcumin inhibits human colon cancer cell growth by suppressing gene expression of epidermal growth factor receptor through reducing the activity of the transcription factor Egr-1. Oncogene 25: 278–287. 16170359 10.1038/sj.onc.1209019

[3] Mohandas KM, Desai DC (1999) Epidemiology of digestive tract cancers in India. V. Large and small bowel. Indian J Gastroenterol 18: 118–121. 10407566

[4] Siegel R, Naishadham D, Jemal A (2012) Cancer statistics, 2012. CA Cancer J Clin 62: 10–29. 10.3322/caac.20138 22237781

[5] Gustin DM, Brenner DE (2002) Chemoprevention of colon cancer: current status and future prospects. Cancer Metastasis Rev 21: 323–348. 12549770 10.1023/a:1021271229476

[6] Gorlick R, Bertino JR (1999) Drug resistance in colon cancer. Semin Oncol 26: 606–611. 10606253

[7] Longley DB, Allen WL, Johnston PG (2006) Drug resistance, predictive markers and pharmacogenomics in colorectal cancer. Biochim Biophys Acta 1766: 184–196. 16973289 10.1016/j.bbcan.2006.08.001

[8] Huo HZ, Zhou ZY, Wang B, Qin J, Liu WY, Gu Y (2014) Dramatic suppression of colorectal cancer cell growth by the dual mTORC1 and mTORC2 inhibitor AZD-2014. Biochem Biophys Res Commun 443: 406–412. 10.1016/j.bbrc.2013.11.099 24309100

[9] Huo HZ, Wang B, Qin J, Guo SY, Liu WY, Gu Y (2013) AMP-activated protein kinase (AMPK)/Ulk1-dependent autophagic pathway contributes to C6 ceramide-induced cytotoxic effects in cultured colorectal cancer HT-29 cells. Mol Cell Biochem 378: 171–181. 10.1007/s11010-013-1608-8 23508272

[10] Schmoll HJ, Stein A (2014) Colorectal cancer in 2013: Towards improved drugs, combinations and patient selection. Nat Rev Clin Oncol 11: 79–80. 10.1038/nrclinonc.2013.254 24445520

[11] Palta M, Czito BG, Willett CG (2014) Colorectal cancer: adjuvant chemotherapy for rectal cancer-an unresolved issue. Nat Rev Clin Oncol 11: 182–184. 10.1038/nrclinonc.2014.43 24642673

[12] Halestrap AP, McStay GP, Clarke SJ (2002) The permeability transition pore complex: another view. Biochimie 84: 153–166. 12022946 10.1016/s0300-9084(02)01375-5

[13] Bonora M, Pinton P (2014) The mitochondrial permeability transition pore and cancer: molecular mechanisms involved in cell death. Front Oncol 4: 302 10.3389/fonc.2014.00302 25478322 PMC4235083

[14] Maldonado EN, Sheldon KL, DeHart DN, Patnaik J, Manevich Y, Townsend DM, et al (2013) Voltage-dependent anion channels modulate mitochondrial metabolism in cancer cells: regulation by free tubulin and erastin. J Biol Chem 288: 11920–11929. 10.1074/jbc.M112.433847 23471966 PMC3636879

[15] Dixon SJ, Lemberg KM, Lamprecht MR, Skouta R, Zaitsev EM, Gleason CE, et al (2012) Ferroptosis: an iron-dependent form of nonapoptotic cell death. Cell 149: 1060–1072. 10.1016/j.cell.2012.03.042 22632970 PMC3367386

[16] Kwon MY, Park E, Lee SJ, Chung SW (2015) Heme oxygenase-1 accelerates erastin-induced ferroptotic cell death. Oncotarget 6: 24393–24403. 26405158 10.18632/oncotarget.5162 PMC4695193

[17] Yang WS, Stockwell BR (2008) Synthetic lethal screening identifies compounds activating iron-dependent, nonapoptotic cell death in oncogenic-RAS-harboring cancer cells. Chem Biol 15: 234–245. 10.1016/j.chembiol.2008.02.010 18355723 PMC2683762

[18] Yagoda N, von Rechenberg M, Zaganjor E, Bauer AJ, Yang WS, Fridman DJ, et al (2007) RAS-RAF-MEK-dependent oxidative cell death involving voltage-dependent anion channels. Nature 447: 864–868. 17568748 10.1038/nature05859 PMC3047570

[19] Jerome-Morais A, Bera S, Rachidi W, Gann PH, Diamond AM (2013) The effects of selenium and the GPx-1 selenoprotein on the phosphorylation of H2AX. Biochim Biophys Acta 1830: 3399–3406. 10.1016/j.bbagen.2013.03.010 23518201 PMC3668444

[20] Qiu Y, Yu T, Wang W, Pan K, Shi D, Sun H (2014) Curcumin-induced melanoma cell death is associated with mitochondrial permeability transition pore (mPTP) opening. Biochem Biophys Res Commun 448: 15–21. 10.1016/j.bbrc.2014.04.024 24735534

[21] Ji C, Yang B, Yang Z, Tu Y, Yang YL, He L, et al (2012) Ultra-violet B (UVB)-induced skin cell death occurs through a cyclophilin D intrinsic signaling pathway. Biochem Biophys Res Commun 425: 825–829. 10.1016/j.bbrc.2012.07.160 22892127

[22] Sommer K, Peters SO, Robins IH, Raap M, Wiedemann GJ, Remmert S, et al (2001) A preclinical model for experimental chemotherapy of human head and neck cancer. Int J Oncol 18: 1145–1149. 11351243 10.3892/ijo.18.6.1145

[23] Bruzzese F, Di Gennaro E, Avallone A, Pepe S, Arra C, Caraglia M, et al (2006) Synergistic antitumor activity of epidermal growth factor receptor tyrosine kinase inhibitor gefitinib and IFN-alpha in head and neck cancer cells in vitro and in vivo. Clin Cancer Res 12: 617–625. 16428508 10.1158/1078-0432.CCR-05-1671

[24] Porter AG, Janicke RU (1999) Emerging roles of caspase-3 in apoptosis. Cell Death Differ 6: 99–104. 10200555 10.1038/sj.cdd.4400476

[25] Diessenbacher P, Hupe M, Sprick MR, Kerstan A, Geserick P, Haas TL, et al (2008) NF-kappaB inhibition reveals differential mechanisms of TNF versus TRAIL-induced apoptosis upstream or at the level of caspase-8 activation independent of cIAP2. J Invest Dermatol 128: 1134–1147. 17989734 10.1038/sj.jid.5701141

[26] Pistritto G, Puca R, Nardinocchi L, Sacchi A, D'Orazi G (2007) HIPK2-induced p53Ser46 phosphorylation activates the KILLER/DR5-mediated caspase-8 extrinsic apoptotic pathway. Cell Death Differ 14: 1837–1839. 17627287 10.1038/sj.cdd.4402186

[27] Choi WS, Yoon SY, Oh TH, Choi EJ, O'Malley KL, Oh YJ (1999) Two distinct mechanisms are involved in 6-hydroxydopamine- and MPP+-induced dopaminergic neuronal cell death: role of caspases, ROS, and JNK. J Neurosci Res 57: 86–94. 10397638 10.1002/(SICI)1097-4547(19990701)57:1<86::AID-JNR9>3.0.CO;2-E

[28] Chen B, Xu M, Zhang H, Wang JX, Zheng P, Gong L, et al (2013) Cisplatin-induced non-apoptotic death of pancreatic cancer cells requires mitochondrial cyclophilin-D-p53 signaling. Biochem Biophys Res Commun 437: 526–531. 10.1016/j.bbrc.2013.06.103 23845906

[29] Zhao LP, Ji C, Lu PH, Li C, Xu B, Gao H (2013) Oxygen glucose deprivation (OGD)/re-oxygenation-induced in vitro neuronal cell death involves mitochondrial cyclophilin-D/P53 signaling axis. Neurochem Res 38: 705–713. 10.1007/s11064-013-0968-5 23322110

[30] Clarke SJ, McStay GP, Halestrap AP (2002) Sanglifehrin A acts as a potent inhibitor of the mitochondrial permeability transition and reperfusion injury of the heart by binding to cyclophilin-D at a different site from cyclosporin A. J Biol Chem 277: 34793–34799. 12095984 10.1074/jbc.M202191200

[31] Sullivan PG, Thompson MB, Scheff SW (1999) Cyclosporin A attenuates acute mitochondrial dysfunction following traumatic brain injury. Exp Neurol 160: 226–234. 10630207 10.1006/exnr.1999.7197

[32] Zhou C, Chen Z, Lu X, Wu H, Yang Q, Xu D (2015) Icaritin activates JNK-dependent mPTP necrosis pathway in colorectal cancer cells. Tumour Biol. 10.1007/s13277-015-4134-3 26427664

[33] Chen L, Li X, Liu L, Yu B, Xue Y, Liu Y (2015) Erastin sensitizes glioblastoma cells to temozolomide by restraining xCT and cystathionine-gamma-lyase function. Oncol Rep 33: 1465–1474. 10.3892/or.2015.3712 25585997

